# Environmental Determinants of Woody Plant Diversity at a Regional Scale in China

**DOI:** 10.1371/journal.pone.0075832

**Published:** 2013-09-26

**Authors:** Hong Qian

**Affiliations:** Research and Collections Center, Illinois State Museum, Springfield, Illinois, United States of America; Utah State University, United States of America

## Abstract

Understanding what drives the geographic variation of species richness across the globe is a fundamental goal of ecology and biogeography. Environmental variables have been considered as drivers of global diversity patterns but there is no consensus among ecologists on what environmental variables are primary drivers of the geographic variation of species richness. Here, I examine the relationship of woody plant species richness at a regional scale in China with sixteen environmental variables representing energy availability, water availability, energy-water balance, seasonality, and habitat heterogeneity. I found that temperature seasonality is the best predictor of woody species richness in China. Other important environmental variables include annual precipitation, mean temperature of the coldest month, and potential evapotranspiration. The best model explains 85% of the variation in woody plant species richness at the regional scale in China.

## Introduction

Species richness varies greatly among different regions across the globe [1]. Understanding what drives the geographic variation of species richness is a fundamental goal of ecology and biogeography. Strong correlations between environmental variables and species richness have been found for many taxa at all spatial scales across the globe, with the highest species richness occurring in warm and wet areas [2]. On this basis, environmental variables have been considered as primary drivers of global diversity patterns by some authors (e.g., [3]–[4]). Numerous environmentally based hypotheses have been proposed to explain the geographic variation of species richness [5]. Environmentally based hypotheses may be grouped into a few broad categories such as energy availability, water availability, energy-water balance, seasonality, and habitat heterogeneity but different studies have frequently found different primary forces determining species richness for the same taxa at different spatial scales in the same or different regions (e.g., [6]). This suggests that a full understanding of the origin of geographic variation in species richness requires many more analyses of the relationship between species richness and environmental factors using species richness data for different taxa documented at different spatial scales in different regions.

Woody plants are an ideal group of organisms for examining the relationship of species richness with environment in general and with climate in particular because (1) plants stay where they are year round (in contrast to animals which may migrate into more favorable sites in winter) and (2) woody plant species are large, compared to herbaceous plants, and many woody plants (particularly trees) expose their stems and buds (with reproductive organs) in air in winter and thus are easier to be damaged by cold winter climate (in contrast to herbaceous plants which can be protected from frost by being annual, by the production of underground buds and stems, or by snow above them during winter time). Thus, it is reasonable to assume that if climatic variables are important forces driving gradients of species richness, as suggested by many authors (e.g., [7]–[8]), woody plants should be one of the best groups of organisms for examining the role of climate in shaping the geographic variation of species richness.

Here, I relate woody plant species richness in provincial floras in China to sixteen environmental variables in order to determine which environmental variable or a group of environmental variables is best to explain the geographic variation of woody plant species richness at a regional scale. China is an ideal region for studying species richness – environment relationships for several reasons. First, China is much richer in woody plant species than any other climatically similar regions in the world. For example, within temperate latitudes, the mesic forests of eastern Asia have three times more tree species than those in eastern North America and six times more than those in Europe [1]. Second, China covers a wide range of latitudes and longitudes and has a wide range of variation in climate (e.g., from tropical rain forest northeastward to boreal forest and northwestward to desert), which makes China greatly suitable for testing the relationships between species richness and environment. Third, unlike Europe and North America that were covered by huge ice sheets during Pleistocene glaciations, which have had noticeable effects on current species richness patterns [9], China was largely free of Pleistocene glaciations [10] and, therefore, the degree to which species recolonization after the Last Maximum Glaciation is in equilibrium with current climate is potentially higher in China than in many other parts of the Northern Hemisphere [11].

## Materials and Methods

### Plant data

The national checklist of woody and semiwoody (e.g., subshrub and shrublet) plants in China was initially compiled based the English-language *Flora of China* [12] (also see www.efloras.org/) for seed plants (gymnosperms and angiosperms) and the *China's node of Species 2000* (www.sp2000.cn/joaen/) for pteridophytes (the volumes of the *Flora of China* for pteridophytes have not been published). Woody plant taxa that were included in other national, regional, or provincial floras of China but were missed by the above-mentioned two sources and new taxa and new distributional records for the flora of China that were documented after the publication of their families in the *Flora of China* have been added to the national checklist. The presence or absence of each taxon in each of China's provinces (including autonomic regions) was documented based on published sources, which include, but are not limited to, national floras (e.g., *Flora Reipublicae Popularis Sinicae* [13]; Sylva Sinica [14]; *Higher Plants of China* [15]), regional and provincial floras (e.g., *Flora of Anhui* [16]), and local (primarily nature reserve) floras and checklists. Municipalities were combined with their adjacent provinces (specifically, Beijing and Tianjin combined with Hebei Province, Shanghai combined with Zhejiang Province, Chongqing combined with Sichuan Province, and Hong Kong, Macau, and Shenzhen combined with Guangdong Province). As a result, 28 province-level floras were used in this study (Table 1). The nomenclature of woody plant names in different sources was standardized based on the *Flora of China* for seed plants and the *China's node of Species 2000* for pteridophytes. For those species which possess taxa at an infraspecific rank (e.g., subspecies, variety, and forma), provincial distributions at the species rank were a combination of provincial distributions of both species and their infraspecific taxa. Exotic species were excluded from this study.

**Table 1:** Geographic information (midpoint values of latitude and longitude, maximum elevation, and area) and the numbers of woody and semiwoody plant species in each of China's provinces.

Province	Lat. (°)	Long. (°)	Elev. (m)	Area (km^2^)	Woody	Semiwoody
Anhui	31.5	117.5	1873	139900	1245	71
Fujian	25.5	118.0	2158	123103	2067	131
Gansu	37.7	100.5	5798	455000	1411	155
Guangdong	23.0	113.5	1879	199498	3243	225
Guangxi	23.0	107.8	2142	236000	4400	308
Guizhou	26.9	106.6	2900	176400	3193	200
Hainan	19.2	109.8	1867	33900	2249	175
Hebei	37.5	117.0	2870	219501	658	75
Heilongjiang	47.0	127.5	1712	463600	350	47
Henan	34.5	115.3	2192	167000	1308	82
Hubei	31.2	112.0	3105	187516	2099	127
Hunan	27.5	112.0	2120	210490	2396	145
Jiangsu	32.5	119.0	642	106000	1202	85
Jiangxi	27.5	116.0	2120	164800	2089	125
Jilin	43.0	126.0	2691	187000	331	44
Liaoning	42.0	122.0	1500	151000	488	60
Neimonggu	44.0	115.0	2034	1150000	430	116
Ningxia	37.3	106.0	3556	66400	456	79
Qinghai	35.5	96.3	6860	720000	559	103
Shaanxi	36.0	108.0	3767	195800	1483	109
Shandong	37.5	118.5	1546	153300	594	41
Shanxi	37.5	112.0	3058	157100	696	75
Sichuan	30.0	105.0	7558	569000	3496	272
Taiwan	23.8	121.0	3950	35760	1377	135
Xinjiang	42.0	84.9	8611	1646797	454	205
Xizang	32.0	90.0	8848	1221599	2532	255
Yunnan	25.2	101.5	6740	436208	6216	444
Zhejiang	29.1	120.6	1857	101787	1662	92

### Environmental data

I related species richness to sixteen environmental variables as follows: (1) mean annual temperature (TEM, °C), (2) mean temperature of the coldest month (TEMmin, °C), (3) mean temperature of the warmest month (TEMmax, °C), (4) annual temperature range (TEMvar; i.e., the difference between TEMmax and TEMmin), (5) the standard deviation of mean monthly temperature (TEMsd), (6) annual precipitation (PREC, mm), (7) summer precipitation (PRECsum, mm; i.e., the sum of monthly precipitation from May through August), (8) annual precipitation range (PRECvar, mm; i.e., the difference in precipitation between the wettest and driest months), (9) the standard deviation of mean monthly precipitation (PRECsd), (10) annual actual evapotranspiration (AET, mm), (11) annual potential evapotranspiration (PET, mm), (12) water deficit (WD, measured as the difference between PET and AET [17]), (13) moisture index (MI, measured as the ratio of AET over PET; [18]), (14) the range of mean annual temperature within a province (TEMrange, °C), (15) the range of annual precipitation within a province (PRECrange, mm), and (16) the range of elevation within a province (ELEVrange, m). These environmental variables were grouped into five broad categories representing energy availability, water availability, energy-water balance, seasonality, and habitat heterogeneity (Table 2). These variables are associated with the diversity and large-scale distributions of plants at a wide range of spatial scales, including regions, continents, and globe (e.g., [19]–[23]). Although other environmental variables might also influence geographic distributions of plants, I included all major environmental variables used in previous studies addressing species diversity in a macroecological context and some of the variables included in the present study are strongly correlated with those that were not included in this study. For example, AET has been used as a surrogate of net primary productivity (NPP; [24]), which has been used in some other studies on large-scale species richness. Data for temperature and precipitation were obtained from the CRU Global Climate Dataset developed by the Climatic Research Unit [25]; AET and PET were obtained from the Global Evapotranspiration and Water Balance Data Sets developed by Ahn & Tateishi [26]. In both datasets, data were documented at a resolution of 0.5 degrees in latitude and longitude. I assigned each half-degree pixel to a province according to the latitude and longitude of each pixel's midpoint. A total of 3800 pixels were located in the 28 provinces in China. Averages of the variables for each province were calculated (Table S1), and these averages were used to represent average environmental conditions of the provinces.

**Table 2:** Mean, minimum, and maximum values of environmental variables for China (summarized from the 28 provinces).

Variable	Minimum	Maximum	Mean
**(1) Energy availability**			
TEM	−2.44	23.68	11.50
TEMmin	−22.08	17.88	−1.47
TEMmax	8.58	28.32	23.00
PET	541.38	1425.88	905.81
**(2) Water availability**			
PREC	121.48	2075.55	955.16
PRECsum	64.48	1132.55	548.23
WD	8.12	555.15	169.19
MI	0.16	0.99	0.78
**(3) Energy-water balance**			
AET	101.21	1294.91	736.61
**(4) Seasonality**			
TEMvar	9.85	42.30	24.47
TEMsd	3.68	15.60	9.00
PRECvar	20.38	309.25	165.49
PRECsd	7.21	106.15	57.05
**(5) Habitat heterogeneity**			
ELEVrange	642.00	8765.00	3269.67
TEMrange	1.30	32.90	10.17
PRECrange	193.00	2006.00	540.25

### Data analysis

I used ordinary least squares (OLS) regression analysis to evaluate the power of each of the 16 environmental variables in explaining the geographic variation of species richness across China based on adjusted coefficient of determination (*R* ^2^_adj_). In addition, I built models with various combinations of different environmental variables to examine the relationship between species richness and environmental variables and used the Akaike information criterion corrected for spatial autocorrelation (AIC_c_; [17]) to evaluate model performance [27]. Specifically, for each of the five groups of environmental variables, I selected at least one variable that was best in explaining the variation in species richness (see more in the Results section). I log_10_-transformed species richness to improve normality. When *P*-values were used to determine whether a regression is statistically significant after accounting for spatial autocorrelation, I used Dutilleul's [28] method to correlate the observed and estimated species richness and to test for the statistical significance of the regression based on geographically effective degrees of freedom [29]. Infraspecific taxa and semiwoody plants were excluded from the analyses.

Area varies among the provinces (Table 1). To determine whether area should be included in the analyses as a covariate, I assessed the amount of the variation in species richness that could be explained by area. Because only 1.2% of the variation in species richness was explained by area (both log_10_-transformed) and because the relationship was not significant (*P* = 0.575), area was not included in final analyses.

## Results

China harbors 14,019 taxa of woody or semiwoody plants at the species and lower ranks (autonyms of species, e.g. *Acer pictum* ssp. *pictum*, were not tallied). When only woody plants at the species rank were considered, China possesses 10,989 woody plant species, of which 17, 199, and 10,773 are pteridophytes, gymnosperms, and angiosperms, respectively. The number of woody and semiwoody plant species in a province varies greatly among the 28 provinces (Table 1). On average, each province possesses 1738.7±1397.7 (SD) and 142.2±92.4 species of woody and semiwoody plants, respectively.

Of the four variables of energy availability, PET explained the largest amount of the variation (67.4%) in species richness and was followed by TEMmin (Table 3). Of the four water availability variables, PREC explained the largest amount of the variation (49.4%) in species richness (Table 3). The water-energy balance variable (i.e., AET) explained 48.5% of the variation in species richness. The two variables of temperature seasonality explained nearly the same amount of the variation in species richness and they each explained about twice as much variation in species richness as either variable of precipitation seasonality (Table 3). Of the three variables of habitat heterogeneity, PRECrange was the best explanatory variable but explained only about 10% of the variation in species richness (Table 3).

**Table 3:** Adjusted coefficient of determination (*R* ^2^_adj_) of the linear and quadratic regressions of the log_10_ species richness of woody plants against each environmental variable (see Methods for full names of variables).

Variable	Linear	Quadratic
**(1) Energy availability**		
TEM	0.407 (+)	0.416 (+, +)
TEMmin	0.606 (+)	0.603 (+, −)
TEMmax	0.061 (+)	0.153 (−, +)
PET	0.542 (+)	0.674 (+, −)
**(2) Water availability**		
PREC	0.429 (+)	0.494 (+, −)
PRECsum	0.428 (+)	0.430 (+, −)
WD	0.146 (−)	0.114 (−, −)
MI	0.228 (+)	0.209 (−, +)
**(3) Energy-water balance**		
AET	0.485 (+)	0.468 (+, −)
**(4) Seasonality**		
TEMvar	0.704 (−)	0.707 (−, −)
TEMsd	0.705 (−)	0.705 (−, −)
PRECvar	0.283 (+)	0.259 (+, −)
PRECsd	0.361 (+)	0.338 (+, −)
**(5) Habitat heterogeneity**		
ELEVrange	0.033 (+)	0.005 (+, −)
TEMrange	0.002 (+)	0.004 (−, +)
PRECrange	0.106 (+)	0.100 (+, −)

A sign in parentheses indicates a positive (+) or negative (−) relationship (the second sign in a quadratic regression is for the quadratic term).

**Figure 1:**
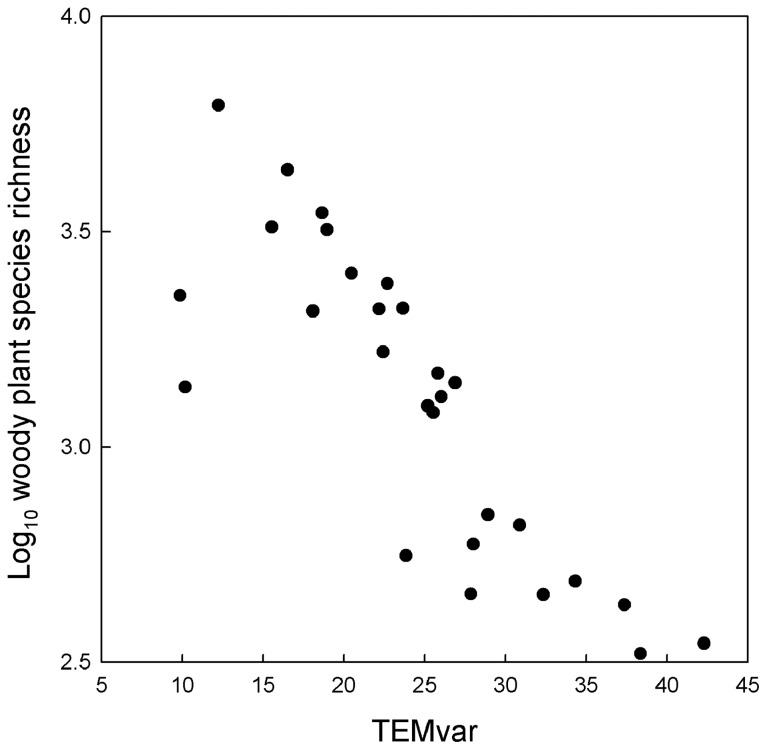
The relationship between woody plant species richness and temperature seasonality (TEMvar) for the provincial floras of China.

The results presented in Table 3 suggested that PREC, PREC^2^, TEMvar, PET, PET^2^, and TEMmin from three of the five groups of environmental variables were among the major determinants of species richness with TEMvar being the best one (Fig. 1). I included these variables and two variables representing the other two groups of environmental variables (i.e., AET and PRECrange) to build regression models representing all possible combinations of these eight independent variables. Of the total number of 255 regression models, the best regression model included PREC, PREC^2^, TEMvar, and PRECrange, based on AIC_c_ (−19.58; Table 4). This regression model explained 85% of the variation in species richness. ΔAIC_c_ between this model and the next two best models was <1 (Table 4), suggesting that these three models are equally good.

**Table 4:** Adjusted coefficient of determination (*R* ^2^_adj_) and Akaike information criterion corrected for spatial autocorrelation (AIC_c_) for the four best fit models of all possible models resulting from various combinations of PREC, PREC^2^, TEMvar, PET, PET^2^, TEMmin, AET and PRECrange (see Methods for full names of variables).

Model	Predictors in model	*R* ^2^_adj_	AIC_c_	ΔAIC_c_
1	PREC, PREC^2^, TEMvar, PRECrange	0.852	−19.58	0.00
2	PREC, PREC^2^, TEMvar	0.839	−19.21	0.37
3	PREC, PREC^2^, TEMvar, PET	0.847	−18.63	0.95
4	PREC, PREC^2^, TEMvar, AET	0.846	−18.46	1.13

All models were significant (P<0.05) after accounting for spatial autocorrelation.

## Discussion

China is one of the richest countries in the world in terms of species diversity of plants [30]. Understanding the status of woody plant species richness within the country, geographic variation in woody plant species richness across the country, and the relationships between woody plant species richness and environments in the country is critical to understanding the origin and maintenance of geographic patterns of plant diversity in general and woody plant diversity in particular in the world. Fang *et al*.'s [31] atlas is the first attempt to compile a complete species list of native woody plants in China and to document the geographic distributions of each woody plant species within China. Their atlas includes 11,405 taxa, which include both species and infraspecific ranks (e.g., subspecies, variety, forma, and cultivar) and include woody, semiwoody, and some herbaceous plants (e.g., *Ajania nitida* C. Shih). However, over 2600 taxa of woody or semiwoody plants native to China were not included in their atlas. These missing taxa from their atlas include trees, shrubs, and lianas. For example, their atlas includes 1175 genera; for the genus *Acer* alone, twelve tree taxa native to China were included in the *Flora of China* [12] but were not included in Fang *et al*.'s atlas, which are *A. acuminatum* Wall. ex D. Don, *A. buergerianum* var. *formosanum* (Hayata ex H. Lév.) Sasaki, *A. buergerianum* var. *horizontale* Metcalf, *A. calcaratum* Gagnep., *A. chunii* ssp. *dimorphophyllum* W.P. Fang, *A. cordatum* var. *dimorphifolium* (F.P. Metcalf) Y.S. Chen, *A. duplicatoserratum* Hayata, *A. kuomeii* W. P. Fang & M. Y. Fang, *A. pictum* Thunb., *A. serrulatum* Hayata, *A. tutcheri* var. *shimadae* Hayata, *A. yangbiense* Y. S. Chen & Q. E. Yang (see www.efloras.org). It is unlikely that nomenclatural standardization is a major cause for the difference in the number of woody and semiwoody taxa between their and my databases because both databases followed the *Flora of China* for botanical nomenclature. Nevertheless, five of the 21 volumes of the *Flora of China* for seed plants were published after the publication of Fang *et al*.'s atlas, which may have, to some degree, caused the incompletion of their checklist of woody plants in China. Furthermore, they claimed that exotic species were excluded from their atlas, but in fact numerous exotic species have been found in their atlas. For example, *Robinia pseudoacacia* L. is a tree species native only to North America and was introduced into China and many parts of the world [12] but this species was included in their atlas as a native species to China. Similarly, *Cytisus scoparius* (L.) Link is a shrub species native to western and central Europe and was introduced into China and many parts of the world [12] but this species was also included in their atlas as a native species to China. Thus, the 11,405 taxa included in Fang *et al*.'s atlas represent a substantially incomplete woody flora of China on one hand and a mixture of native and exotic species on the other hand.

Using the data published in Fang *et al*. [31], Wang *et al*. [32] related woody species richness in 2500-km^2^ quadrats across China to various environmental variables and found that the mean temperature of the coldest quarter is the most important environmental determinant of woody plant species richness in China, which is inconsistent with the result of the present study. However, it is uncertain the degree to which the result of my study can be compared with that of their study partly because the two studies examined the relationship between woody plant species richness and environments at different spatial scales and partly because their study may be substantially biased by several factors. First, as discussed above, over 2600 taxa of woody plants native to China were not included in their study and the data that they used was a mixture of native and exotic species. Second, woody plant species lists for each of their 2500-km^2^ quadrats were generated based on species lists of woody plants for each of the 2408 counties of China. However, because few counties in the mainland of China have been botanized with the aim of generating complete species lists, woody plant species lists are presumably very incomplete for most, if not all, of the counties in their database [33], which would have in turn resulted in substantial underestimates of woody plant richness in their 2500-km^2^ quadrats. Wang *et al*. reported that each 2500-km^2^ quadrat has, on average, 358 taxa of woody plants. However, the average number of woody taxa is 713 in 78 local floras (including reserve and non-reserve localities) widely spread across China, despite that the average area of these local floras is only 725 km^2^ (i.e., about 30% as large as a 2500-km^2^ quadrat). This suggests that woody plant taxa in each of their 2500-km^2^ floras include, on average, far less than 50% of all woody plant taxa in the quadrat. Third, for some taxa in Fang *et al*.'s atlas, county-level distributions were generated not based on actual distributions of these taxa; instead, they were estimated based on the similarity in topographic and climatic conditions between those counties with records of actual distributions of the species and those counties without records of actual distributions. Because native distributions of many species do not cover all of environmentally suitable sites (e.g., Boucher-Lalonde *et al*. [34] found that geographic ranges of species are always entirely surrounded by unoccupied but apparently suitable climates and tree species in North America occupy on average only 29% of their climatic niche) and because disjunct distribution patterns of many species are driven by historical factors [10], rather than by environmental factors, accordingly, including distributions of species estimated based on environmental conditions would have presumably made the distributions in Fang *et al*.'s atlas an artifact to some degree. Using such data in analyses of relating species richness to environmental factors in order to seek for determinants of geographic patterns of species richness would result in biased conclusions due to the issue of circularity (i.e., generating species distributions based on environmental conditions and then relating the generated species distributions to environmental conditions to build the relationship between species richness and environmental conditions). Thus, the data that were published in Fang *et al*.'s atlas and were used in Wang *et al*. [32] are not appropriate for any analyses addressing issues that require reasonably complete species lists based on native distributions, including analyses examining the relationship between species richness and environments.

My study showed that woody plant species richness in China varies greatly along environmental gradients at the regional scale examined. For example, woody plant species richness in Yunnan Province, which is located in a tropical and subtropical region, is about eighteen times higher than that in Heilongjiang Province, which is located in a temperate and boreal region, despite the fact that the two provinces are similar in area (Table 1). My study also showed that of all environmental variables examined, temperature seasonality is the strongest determinant of woody plant species richness at the provincial scale in China. This finding is consistent with those of several previous studies. For example, Raes *et al*. [35] found that the variable explaining most of the variance in plant species richness in Borneo is temperature seasonality; Wiens *et al*. [36] also found that temperature seasonality is the most important climatic variable driving distributions of treefrogs in the New World. This finding is consistent with the hypothesis that climatic seasonality influences the geographic variation of species richness by altering the length of growing season for plants [8] and/or the allocation of energy use of individuals [37]. O'Brien *et al*. [38] found that of the eighteen environmental variables examined, mean annual precipitation was most strongly correlated with woody plant diversity in 25,000-km^2^ quadrats in southern Africa. They did not include temperature seasonality but included the intra-annual range of PET (i.e., maximum monthly PET minus minimum monthly PET) as a measure of thermal seasonality in their study. However, six of the eighteen environmental variables included in their study are more strongly correlated with woody plant species diversity than thermal seasonality. With the same southern African data, O'Brien *et al*. [3] showed that their best model with four explanatory variables explained 80% of the variation in woody plant species diversity. The fact that the best model in my study explained about 5% more variation in woody plant species diversity for China than their best model for southern Africa may be partly because my study used a larger spatial scale. Previous studies have shown that for the same group of organisms in the same study system, environmental variables explain more variation in species richness in sampling units at a larger scale than those at a smaller scale [39]. Currie & Paquin [7] found that of the eighteen environmental variables examined in their study, AET is most strongly correlated with tree species richness in sampling units of 51,000–71,000 km^2^ in North America. The fact that different environmental factors have been found to be correlated with woody plant species richness in different regions may suggest that in addition to the effect of spatial scale, regional and historical factors have played a role in determining geographic variation in species richness, which may influence the relationship between species richness and environments differently in different regions [10].

Habitat heterogeneity, particularly elevation range, has been considered as a major determinant of species richness in previous studies. For example, O'Brien *et al*. [3] showed that elevation range explains 33.6% of the variation in woody plant species richness in southern Africa (*r* = 0.58 between the two variables); Moore *et al*. [40] found that elevation range explained 34.8% of the variation in vertebrate species richness in 2° by 2° (or ∼48,000 km^2^) quadrats in sub-Saharan Africa (*r* = 0.59 between the two variables); Kreft & Jetz [41] showed that elevation range explained 24.8% of the variation in vascular plant richness in geographic units of varying areas (ranging from 10 km^2^ to 300,000 km^2^) across the world. In contrast, elevation range explained only about 3% of the variation in woody plant species richness in the provincial floras of China. However, this does not necessarily mean that habitat heterogeneity is not an important factor influencing geographic variation in woody plant species richness at the regional scale examined. The fact that little variation in species richness was explained by measures of habitat heterogeneity in my study may be largely because there is not much variation in habitat heterogeneity among the 28 provinces. For example, elevation range is greater than 1500 m and 2000 m for 27 and 19, respectively, of the 28 provinces in China, and the mean of elevation ranges for the 28 provinces is 3270 m (±2191 SD). I expect that elevation range would have explained much more variation in woody plant species richness among regional woody floras if there would be substantially more variation in elevation range among the provinces in China.

## Supporting Information

Table S1 **Geographical, species richness, and environmental data used in the analyses presented in this study.**
(CSV)
